# Scheduling in the context of resident duty hour reform

**DOI:** 10.1186/1472-6920-14-S1-S18

**Published:** 2014-12-11

**Authors:** Ning-Zi Sun, Thomas Maniatis

**Affiliations:** 1 Division of General Internal Medicine, McGill University Health Centre; Department of Medicine, McGill University, QC, Canada

## Abstract

Fuelled by concerns about resident health and patient safety, there is a general trend in many jurisdictions toward limiting the maximum duration of consecutive work to between 14 and 16 hours. The goal of this article is to assist institutions and residency programs to make a smooth transition from the previous 24- to 36-hour call system to this new model. We will first give an overview of the main types of coverage systems and their relative merits when considering various aspects of patient care and resident pedagogy. We will then suggest a practical step-by-step approach to designing, implementing, and monitoring a scheduling system centred on clinical and educational needs in the context of resident duty hour reform. The importance of understanding the impetus for change and of assessing the need for overall workflow restructuring will be explored throughout this process. Finally, as a practical example, we will describe a large, university-based teaching hospital network’s transition from a traditional call-based system to a novel schedule that incorporates the new 16-hour duty limit.

## Introduction

There has been a worldwide movement in recent years to reduce the maximum consecutive duration of resident duty hours. This has been fuelled mainly by concerns about resident health and patient safety [1,2]. Although the actual limit on resident duty hours currently differs by both country and jurisdiction [3,4], there is a general trend to gradually move to a model in which the maximum number of consecutive working hours is limited to between 14 and 16 hours. This move represents an important shift from the previous 24- to 36-hour call duty systems. Although some jurisdictions have quickly embraced the change and instated novel call schedules in the context of resident duty hour reform, the 14- to 16-hour limit remains a novel concept to many institutions, making the transition a daunting one for them.

To provide background for this discussion, we will begin by giving an overview of the main types of coverage systems currently used in different jurisdictions, including their characteristics and suitability for various clinical settings and types of residency programs. To this end, we performed a search of the literature and selected descriptive and/or evaluative studies of novel call duty models published within the last 20 years. However, given that the trend toward a 14- to 16-hour limit on consecutive working hours – such as the one outlined by the 2010 Accreditation Council for Graduate Medical Education (ACGME) requirements in the United States [5] – is relatively new, practical descriptions of scheduling systems that fit this requirement are scarce in the published literature.

We will then suggest a practical step-by-step approach to designing, implementing, and monitoring a scheduling system centred on clinical and educational needs in the context of resident duty hour restrictions. We would like to highlight that a number of the recommendations that appear throughout this article, while inspired by the results of our literature review, are based on our experience. Finally, we will describe a large, university-based teaching hospital network’s transition from a traditional call-based system to a novel schedule that integrates resident duty hour restrictions.

## Review of terminology

A challenge in any discussion of duty hour systems is that institutions from different jurisdictions use different terms to describe resident scheduling systems. In this section, we will briefly review the terms most commonly used in North America. We will also use this opportunity to briefly discuss the advantages and drawbacks of each of these duty hour systems.

*Call* is the term used in the traditional 24- to 36-hour duty systems to designate the after-hour period (typically beginning at 5-6 p.m. and continuing until 8 a.m.-12 p.m. on regular weekdays, and from 8-9 a.m. to 8 a.m.-12 p.m. on Saturdays, Sundays, and holidays). The resident(s) assigned to cover this period are said to be *on-call*. The daytime period prior to the start of the on-call period on a regular weekday is often referred to as the *pre-call* period, and the day immediately following the call is the *post-call* day.

Cross coverage is a scheduling strategy in which one or more residents assume the duties of other service(s) in addition to their own. This is frequently used to reduce manpower requirements at times of reduced clinical needs or educational opportunities, and can be incorporated into almost any schedule system, given appropriate consideration of patient care needs and resident abilities. Cross coverage implies that residents provide clinical coverage to patients they are necessarily less familiar with. However, the type of coverage provided is often for emergencies only, which may offset some of the discomfort often associated with cross covering, particularly if these times are well chosen to coincide with lower clinical acuity. Further, the articulation of clear sets of expectations for the cross-covering residents will help them to determine which tasks can appropriately wait until the next day and which need to be addressed immediately. It should be noted that cross coverage is used sparingly by many training programs (e.g., Family Medicine) and tends to work best when combined with effective handovers and the clear articulation of expectations.

### Overview of the types of schedule systems

To contextualize further discussion, we will now review the structure of the traditional 24- to 36-hour call system. In this system, each clinical service or unit is typically staffed by a dedicated resident or team of residents during regular working hours. One or more residents from this pool of trainees are designated as on-call and remain on duty after hours. On the next day, the post-call residents either hand over patients as soon as the daytime team arrives and then leave the hospital or they remain on-site for daytime pedagogical (e.g., morning report) or clinical activities. The duration of consecutive work hours can therefore range from 24 to 36 hours, depending primarily on the length of the post-call period.

The call duty systems that have been described in the literature since the start of the movement toward duty hour reform are all based, in one form or another, on the concept of *floats* versus *shifts*. *Shifts* involve one or a number of residents (more often a single resident) covering a given clinical service or unit in either a sequential (*shifts*) or overlapping (*staggered shifts*) fashion. On the other hand, when used in conjunction with a traditional call system, a *float* system allows for extra coverage during peak service hours or for the relief of residents scheduled to be on-call or who are just finishing their call.

While the shift-based schedule systems have not significantly changed since the introduction of duty hour restrictions, the concept of floats has lost some of its purpose and defining features. Furthermore, as the length of consecutive duty hours shortens, the difference between floats and shifts has become less clear, with the structure of float schedules, with their increasingly shorter periods of consecutive duty, starting to more closely resemble shift work.

In any case, the key difference between float and shift systems concerns manpower distribution. In a shift system, residents are assigned to clinical services or units in a manner that is consistent with the usual division of labour of attending physicians and, often, of nursing teams. In comparison, residents following a float schedule are usually assigned to multiple related services or units in addition to, or in place of, the usual teams responsible for these, and they move (i.e., float) from one service to another in response to clinical needs. Other major differences between float and shift systems include the size of the team (with floats being more amenable to a team of residents, while shifts are more often staffed by a single resident), the use of cross coverage (almost always with floats and less commonly with shifts), and the frequency of rotation between day and night duties (generally more frequent with shifts than with floats). In the model presented, there is necessarily some overlap between these systems.

### Float system

In a *day float system*, resident(s) work only during a specific period during the day and they share the workload of multiple services or units depending on service needs [6-9]. In most models, the float team’s main – and sometimes sole – responsibility is to admit patients, usually during the peak admission time for the given institution. This system helps to alleviate the workload of either the pre-call or post-call residents in the context of the classic 24- to 36-hour call system. Although this system has been useful for a number of programs in the United States in complying with the 2003 ACGME requirements (i.e., an 80-hour week averaged over 4 weeks, with 30-hour shifts and a minimum of 10 hours of rest between shifts) [10], it is no longer a viable standalone option given the limitation of the maximal consecutive work length to 14 to 16 hours. Nonetheless, the key concept behind day float – adding a resident or team to cover periods of peak service – has been adopted by a number of scheduling systems and is a valuable concept to keep in mind when designing schedules. In this system, the day float team is generally available to participate in daytime pedagogical activities.

The *night float system* employs a similar concept [6,11-14]. Residents work only during the night and have no other daytime pedagogical or clinical responsibilities. The precise start and end times of the work period described in the literature varies, but is generally from 6-10 p.m. until 6-10 a.m. Because, in some institutions, night float residents may not start working before 8 to 10 p.m., this system is often coupled with a system of *evening call duty* in which designated residents stay on duty after a regular work day until the arrival of the night float team to provide coverage and effective handover. Of note, this evening coverage has been referred to by various names in the literature, including *half-call*[11] or *call duty*[15], in jurisdictions where the traditional 24-hour call has been abolished. In our experience, the night float team has very limited exposure to daytime academic activities, and there are limited opportunities for direct observation of residents by attending physicians. As such, this has implications for attending-directed teaching and resident evaluation.

In their discussion of the advantages and drawbacks of around-the-clock staffing of intensive care units by attending physicians, Kerlin and colleagues have alluded to the potential benefit to resident supervision and teaching of the overnight presence of an attending physician [16]. However, they also cautioned against the potential decrease in resident autonomy and experiential learning. In addition, large-scale implementation of 24-hour faculty staffing as described by Kerlin and colleagues will undoubtedly increase work pressure on attending physicians and may lead to burnout. Our experience shows that, as an alternative to overnight attending physician staffing, near-peers may successfully take on the role of teaching and supervision, such that senior residents supervise more junior residents and provide feedback on their performance, and vice versa.

### Shifts

A *simple shift-based system* usually involves teams working non-overlapping 8- to 12-hour shifts with handover of the entire service at change of shift. This system – with one to two handover(s) in a 24-hour period – is often used by Emergency Medicine programs because these programs have less predictable peak service periods with a high number of cases throughout the 24 hours, as well as rapid patient turnover.

In *staggered shifts* – a variation of the simple shift system – there is significant overlap (generally two hours or more) between the various shifts [17,18]. This system is often favoured when continuity of care is a priority, such as on services or units where patients are either critically ill or have more complex problems. Staggered shifts may also be used to better match manpower to service needs. In this case, the shifts are usually scheduled so that they are staggered over the period of peak service. The overlap period between the departing and arriving residents is important because it allows for the handover of detailed information; residents round together on acutely ill patients and are able to provide feedback about decisions made by the outgoing residents.

### What we know about the effects of day–night reversal

Scheduling in the context of resident duty hour reform requires an understanding of the effects on residents of switching between daytime and nighttime duty, as this is a requirement of most scheduling systems. Issues that need to be considered are the direction of rotation, the number of night shifts or third shifts (in an eight-hour per shift system) in a row, the frequency of shift rotation, and the shift length.

Studies on circadian rhythms have shown that the human body is physiologically more able to delay than to advance sleep-making adjustments; that is, *clockwise* (*forward* or *phase delay*) rather than *counter-clockwise* (*backward* or *phase advance*) rotations are easier to adjust to [19-23]. It is also much easier to adjust to a two hour rather than a four hour or more delay in sleep [21].

While there is still debate as to the optimal number of consecutive night shifts as it relates to workers’ well-being and performance, two strategies have emerged in the literature [19,24,25]. One approach is to schedule a year’s worth of night shifts consecutively over four to eight weeks. The advantage of this approach is that it optimizes alertness by allowing for complete adjustment to the night schedule. However, it is important that the residents continue to follow the reversed schedule during any off days between night shifts, so as not to lose the adaptation. When using this strategy, the impact on residents’ family and social life must be acknowledged and addressed through proper education of all parties. The second approach is to assign the fewest number of consecutive night shifts to each individual resident, thereby avoiding having to reverse their circadian rhythms altogether. Both strategies generally avoid scheduling four to seven nights in a row because, physiologically, it typically takes four to seven days to fully achieve an eight-hour phase shift [24,26,27].

It seems to be problematic to rotate work time once a week or more frequently [21,22,28]. This limits the potential use of isolated night shifts (i.e., the second strategy explained above) when a group does not have the critical number of physicians to allow for proper shift spacing [24]. It has been suggested that shift rotation should ideally not happen more often than monthly, particularly when a long stretch of consecutive night shifts is scheduled (i.e., the first strategy explained above) [21]. The number of consecutive night shifts and the frequency of shift rotation are therefore two aspects of schedule design that must be considered in tandem with and in relation to the availability of human resources.

Shorter shifts (e.g., 8 to 10 hours) are generally preferred over longer ones (e.g., 12 to 16 hours) from the perspectives of fatigue accumulation and ease of adaptation after shift rotation [19,21,22,24,27]. This is particularly true when patient acuity is high and when it is unlikely that a resident will get two to three hours of sleep during a night shift [19,29]. This being said, one must be aware that there will be an increased number of handovers with these shorter shift lengths. The potential for information loss and adverse patient outcomes with frequent handovers needs to be recognized and addressed explicitly [30]. More information on this can be found elsewhere in this supplement [31]. When longer shifts are used, strategic napping may help alleviate resident fatigue [24]. Because it is easier to work longer shifts during the day than at night, another strategy is to split the 24 hours into longer day (and evening) shifts and shorter night shifts [19].

In summary, the length of the shifts, the number of consecutive days with overnight duty, and the frequency and direction (clockwise versus counter-clockwise) of shift rotation are all important elements to consider when creating a new duty schedule. This applies not only to shift-based systems, but also to float-based systems, as the length of consecutive working hours in the latter may be shorter with the new regulations.

## A practical step-by-step approach to designing, implementing, and monitoring a scheduling system in the context of resident duty hour reform

Table 1 provides a summary of the seven-step approach outlined below.

**Table 1:** Items to consider with respect to the provision of safe and effective clinical care

**Human resource considerations: numbers** • Estimate the number of residents rotating through the service(s)/unit(s) in question at any given time • Estimate the number of residents rotating through other services that can be called upon to help with the coverage of the service(s)/unit(s) in question • If needed, determine the type and amount of non-resident human resources needed to address gaps in coverage • Anticipate and plan for resident illness or other last-minute absences; consider having a backup schedule
**Human resource considerations: responsibilities** • Identify and characterize the main tasks to be performed both within and outside of regular working hours in terms of complexity, acuity, and frequency • Identify the time of peak services and estimate the minimum number of residents required to handle the caseload in a safe and effective fashion • Consider the most resource-intensive tasks done at night and estimate the minimum number of residents required to manage these tasks in a safe and effective fashion
**Allocation of responsibilities across the day and night** • Review the workflow over the entire 24 hours of the day and 7 days of the week • Characterize the types of tasks to be performed at night • Determine how many of these tasks are medical emergencies and how many are administrative, psychosocial, or chronic-care related (i.e., Do the tasks require the immediate availability of an in-house resident or can they wait?) [40] • Take note of tasks that may be better taken care of by the day team that knows the patients best • Restructure care provision based on the above, and then re-estimate your human resource needs • Estimate the average caseload at night both in terms of volume and acuity (i.e., Is cross coverage of two or more services by the same team a viable option?) • Estimate the frequency by which the most resource-intensive tasks (e.g., response to trauma, code blue) occur. If they occur only occasionally, consider negotiating with other services/units to combine their teams with yours to deal with these situations. This will allow you to staff your team based on the requirements of other more-commonly occurring tasks, while allowing some flexibility during times of increased need • Determine the optimal length of shifts for the service/unit in question (i.e., balance the effect of fatigue from longer shifts with the impact on continuity of care from shorter shifts) • Estimate the length of handovers as well as their complexity (i.e., Is it best carried out in person, by phone, or in writing?) [31] • Consider measures to optimize information transfer at handovers (e.g., having more senior residents (fellows) or attending physicians present by phone or video-conference) to focus attention on the patients who are most ill

### Step 1 – Determine why the change is being considered

Before proceeding to work on detailed scheduling systems for your hospital or clinical service, it is essential to understand the impetus for the change (i.e., mandated by regulatory body or a voluntary quality improvement initiative) and to acknowledge the organizational and psychosocial impact of this culture shift, including concerns about perceived loss of both professionalism and resident learning opportunities [32-36]. Surgical residencies have faced significant challenges in adopting the new duty hour requirements, with logistics and a perceived loss of professionalism often being cited as barriers to the successful transition to a new duty hour system [37,38]. Efforts aimed at addressing faculty perceptions and resident professional identity were particularly helpful in these instances [39]. Other frequently cited areas of concern include lost learning opportunities and disruption to continuity of care. All of these issues will need to be considered early on in the change process so that plans for addressing them are built into the proposed plan for change.

### Step 2 – Decide who should be involved in the transition

In our experience, the traditional call system is close to the core of the culture of medicine: it is considered to be a necessary experience that allows young trainees to become attending physicians. Given this, we recommend that a significant effort be made to plan who will be involved in various stages of the change process. The main stakeholders should be identified and involved in the process as early as possible, not only to help design a system that meets clinical and educational needs, but also to lead this important culture shift during the implementation phase. Tempting though it may be to include only those stakeholders who are clearly in favour of change, it is prudent to include individuals with a variety of views so that challenges are identified and addressed early.

As the process unfolds it is important to broaden involvement outside of the select group. This will allow for wider consultation, thereby identifying potential challenges as well as the strengths of proposed models in specific contexts. However, it is important that the major roadblocks be addressed before moving ahead to wider consultation, lest the process be perceived as disorganized or ineffective.

### Step 3 – Clarify terms so everyone is speaking the same language

It is critical to ensure everyone involved in this process is speaking the same language by using terms with definitions that are agreed upon in your jurisdiction. You should first familiarize yourself with the terminology used by your local regulatory bodies (if applicable), particularly if a duty hour reform has been mandated for your institution. If you are implementing a new schedule on a voluntary basis, you may have the flexibility to define the terms yourself.

In addition, if local regulations are already in place in your jurisdiction with respect to length and frequency of resident duty hours, you should review and understand them thoroughly. These regulations may cover any or all of the following:

• Maximum duty hours per week

• Maximum length of continuous work

• Whether the time for handover must be included when calculating the length of continuous work

• Maximum number of consecutive days worked

• Maximum number of consecutive nights worked

• Maximum frequency and number of call duty per time period

• Maximum frequency of weekend call duty per time period

• Maximum frequency of shift rotations (from day to night or vice versa), and whether there are any restrictions on the direction of rotation (i.e., clockwise versus counter-clockwise)

• Minimum rest between or before work periods

• Minimum nap time during long (12 hours or more) shifts

• Whether a distinction on any of the previous considerations is made based on the seniority of the resident (e.g., the 2010 ACGME regulation stipulates that PGY-1 residents can no longer work more than 16 hours per shift, while PGY-2s (and above) can work up to 28 hours, consisting of a 24-hour shift plus 4 hours for transitions) [5]

• Whether there is allowance, on occasion, for work duration beyond the prescribed maximum, as well as the acceptable types of justification for such

### Step 4 – Ask for help from those who have already made the change

Others in your own or neighbouring jurisdictions may have already designed and implemented new call duty systems. It is worthwhile to look for successful models from institutions with clinical services, training programs, and/or call duty regulations similar to yours. It is generally easier to adapt a pre-existing schedule to your needs than to create your own from scratch. If you find a scheduling system that seems particularly interesting, it may be useful to contact the institution in question for more information.

### Step 5 – Develop a model based on your clinical and educational needs

The two major areas for consideration when designing a new schedule are the need to deliver safe and effective patient care and to support a safe and effective pedagogical environment. With respect to patient care, the adequacy of human resources is a major concern. Specifically, if resident work hours are reduced, more residents will be needed to cover the same patients or, alternatively, non-resident coverage will need to be considered (such as physician assistants, nurse practitioners, etc.). While this is an undeniable fact, the increased staffing levels required will vary according to the specific duty hour restrictions being implemented. Some systems may require the development of a one- or two-week block of night floats to cover general medical patients overnight, while others may require an increased resident presence in the evenings or on weekends for certain shifts.

Once you have finished looking at your available resident and other resources, the next challenge is to address the optimization of human-resource utilization 7 days per week and 24 hours per day. Although the emphasis is often put solely on work organization at night, reviewing and redesigning the daytime workflow is sometimes more important to ensure that the night team’s time is spent wisely [40]. It may no longer be appropriate for the day team to leave routine or non-urgent tasks for the night team, who will be less familiar with the patients admitted by the day team. Simply articulating this principle to the health care teams involved often helps them to recognize the importance of this task redistribution.

Table 2 gives a list of some of the items that one should consider to ensure that the new schedule will adequately respond to patient care needs.

**Table 2:** Items of potential interest when planning post-implementation monitoring

**Resident quality of life** • Fatigue • Sleep time • Time spent with family
**Resident education** • Time spent reading • Attendance at teaching activities • Exposure to various procedures/interventions • Performance on standardized tests • Performance on certification exams
**Resident clinical performance and professionalism** • Availability during the night • Development of shift mentality • Quality and nature of interactions with patients and other health care professionals
**Impact on faculty** • Workload • Resident teaching, supervision, and evaluation • Relationships with residents
**Impact on patient care** • Continuity of care • Morbidity and mortality measures • Patient satisfaction • Medication errors • Quality of handovers
**Impact on medical students** • Teaching and evaluation by residents • Patient exposure/caseload • Continuity with their teams

Once you have reviewed the patient care needs of the service(s)/unit(s) to be covered, it is important to analyze the pedagogical and other needs of the residency training program(s) that will be affected by this schedule change. Aspects to consider include resident learning, supervision, and evaluation. The impact of duty hour restrictions on resident morale should also not be overlooked. Specifically, residents may feel isolated if they are coming in for shifts alone at seemingly odd hours, and this sense of isolation can be better managed if residents are paired up in some fashion or come in as a team. It is expected that the organization of academic activities (e.g., rounds, morning reports), as well as the workflow of staff physicians and medical students, will need to be modified in concert with these scheduling changes. These issues are dealt with in detail elsewhere in this supplement [32,35].

Introduction of the new schedule may also affect the scheduling of other activities in the training program. Introduction of one- to two-week rotation blocks (e.g., a night float model with rotation every one to two weeks) can lead to significant fragmentation of other rotations in a residency program. One solution is to consider pairing these one- or two-week blocks with another short rotation to ensure that, combined, they add up to the standard rotation length for the training program. Anticipating the systemic challenges created by various scheduling systems will go a long way to improve resident and faculty buy-in.

Given concerns over resident exposure to an adequate number of cases of various types in the context of resident duty hour reform, it is important to consider the total time worked per week in order to ensure that any proposed schedule allows for an equivalent amount of time spent on duty as compared with past schedules. Of course, the nature of the exposures while a resident is working under the new schedule may not be analogous to past schedules (more or fewer day or night experiences). As such, monitoring the extent of day versus night exposure for residents should be part of the ongoing quality improvement process of the program. This will require particular attention in procedural-based training programs, in which time worked does not necessarily correlate with exposure to specific procedures. This is discussed in more detail elsewhere in this supplement [35].

It may be that you work through the steps outlined above and determine that your current level of resident coverage and duties during the day and night are appropriate for your centre. A practical approach to the actual designing of a schedule is to begin with what is already working at your centre or hospital. If the resident coverage is generally felt to be adequate for patient care and educational needs, then designing a schedule that replicates the number of residents and tasks may be all that is required. Of course, the residents for overnight duties will no longer be drawn from the same pool of daytime residents; the former will be drawn from a new pool, thereby respecting the duty hour restrictions. If your current system is felt to be inappropriately staffed in some areas (either under- or over-staffed), this would be a most opportune time to reallocate resident resources on the basis of local feedback.

Finally, it is important to keep in mind that these new schedules will result in gaps that must be covered. For example, if you have a team that works five nights out of seven, you must also cover the remaining two nights. This can result in loss of exposure to other rotations if residents are pulled away from a rotation in order to cover the gaps. In our experience, implementation of one- to two-week night float blocks has resulted in increased rotation fragmentation. There has also been some institutional reluctance to accept residents for clinical rotations for periods shorter than the traditional four weeks. As a result, consideration must be given to designing one- to two-week rotations that can be used to fill in these gaps (e.g., educational development weeks, research weeks). Of course, the availability of non-resident health care providers (e.g., physician assistants, nurse practitioners) to cover some of these shifts can effectively relieve some of the service pressures associated with the new schedules. Where this is not possible, creative solutions to cover the gaps should be envisaged, such as a *parachute week* in which residents do a series of night shifts across various services to cover any gaps. This would, of course, require coordination of schedules across rotations (and possibly even across hospitals), which can be a complex task.

### Step 6 – Implement and monitor

Once you have designed a new call duty system and fine-tuned it in light of the considerations outlined above, it is time to implement the system and evaluate its impact. Because lack of both buy-in and practice leadership are often important barriers to any large-scale change [41], all affected parties (the service/unit chief/head, attending physicians, program director(s), chief residents, residents, and nursing and auxiliary staff) should be well informed before the schedule takes effect. We recommend that you encourage the local leaders whom you have engaged since the early stage of this process to act as advocates in effecting this culture change. We also recommend that a system of active monitoring and feedback collection be developed and implemented once the schedule is ready. You can choose a phased approach to implementation (e.g., one site or one service/unit at a time) when you roll out the new schedule rather than implementing it simultaneously across multiple services/units and sites. The approach you choose will depend on the resources available for implementation and monitoring at each site.

It is important not to underestimate the impact of these changes and to set a realistic timeline for evaluating and fine-tuning the system. Feedback from residents, staff physicians, auxiliary health professionals, and program directors should be actively sought and reviewed early on. If areas of concern were identified during the creation of the schedule, consider specifically inquiring about these areas in order to see if planned solutions are effective. Make sure to have a mechanism in place for incident-driven review prior to implementation. It is important that you are prepared to act quickly in the face of important unanticipated events and to adapt and fine-tune the new schedule along the way. This being said, while some pitfalls of a new schedule will reveal themselves early on, it may take more time for residents, attending physicians, patients, nurses, and other members of the health care team to fully appreciate the new schedule’s advantages. You should, therefore, consider a longer time frame (e.g., 6 to 12 months) before deciding on the sustainability of the schedule or undertaking major revisions. It is essential to avoid focusing solely on the part of the schedule that seems to be the most problematic. Reviewing the entire workflow throughout the day and night will often reveal broader issues of workload distribution that may be addressed more productively rather than focusing on the details of, for example, a shift start or end time.

### Step 7 – Routine monitoring

Having gone through all of the steps outlined previously, you should be well on your way to developing and implementing a schedule that respects resident duty hour restrictions while ensuring the provision of safe and effective patient care and appropriate resident education. Ongoing measures of quality should continue to be sought from residents, faculty, program directors, patients, nurses, and other members of the health care team, with periodic adjustments made on the basis of this feedback. You can also consider gathering feedback from residents’ spouses.

There is really no one-size-fits-all method or instrument for monitoring and assessing a new duty hour system. If the impetus for implementing the new schedule is a response to a particular need, whether this need has been successfully addressed would obviously be at the centre of post-implementation monitoring. Compliance with any mandated duty hour limits should be tracked, and violations should be evaluated on a case-by-case basis. Beyond these, there are many aspects that could be assessed. Table 3 lists the variables that have been the most frequently identified in the literature.

**Table 3:** Instruments/measures most commonly used in the assessment of a new duty hour system

• Surveys of stakeholders (e.g., residents, faculty, medical students, other health care professionals) [12,42-48] • Epworth Sleepiness Scale [49] • Procedure/operative logbook [50] • Admission/clinical case/operative case counts [51] • Rotation evaluations [50] • Attendance at teaching activities (including conferences) [52] • Standardized in-training tests [17,50] • Certification/qualifying/licensing exams [53] • Patient satisfaction surveys [12] • Patient morbidity and mortality outcomes [17,49,50] • Other patient care measures (e.g., readmission rate, length of stay, adherence to guidelines) [17,49,54] • Statistics on medication errors [55-57]

If you are primarily interested in scientific research, both quantitative (e.g., resident surveys, morbidity and mortality data) and qualitative (e.g., focus group, interviews) methods may be used. However, the latter is usually more time-consuming and may not be suitable for post-implementation monitoring for local quality control purposes. Most authors used a pre–post design either prospectively (i.e., data collection – implementation of new schedule – repeat data collection) or retrospectively (i.e., participants are asked to rate their experience post-implementation and to recall at the same time their experience prior to the new schedule). While the latter is less resource-intensive, it is also prone to recall bias. Another potential study design is a concurrent controlled trial. This type of trial is particularly useful when a new schedule is piloted in one of many similar units, where the units not adopting the change can serve as control groups.

The assessment tools used will depend on the outcomes of interest. Some authors have published detailed descriptions of their assessment instruments, which may be adapted to suit the needs of different residency programs (see Table 4). However, it is not always feasible or necessary to be this comprehensive with post-implementation monitoring. We would suggest focusing efforts on a limited number of outcomes, looking at selected resident and patient care variables.

**Table 4:** Summary of the seven-step approach to designing and implementing a new duty hour system

**Step 1 – Determine why the change is being considered** • Understand the impetus for the change (i.e., mandated versus voluntary) • Acknowledge the organizational and psychosocial impact of this important culture shift **Step 2 – Decide who should be involved in the transition** • Identify and involve the main stakeholders **Step 3 – Clarify terms so everyone is speaking the same language** • Review definitions of key terms • Review local regulations **Step 4 – Ask for help from those who have already made the change** • Identify best practice models from care units or services similar to your own • Look for schedule models that can be adapted to your needs **Step 5 – Develop a model based on your clinical and educational needs** • Consider your clinical needs, including manpower requirements, workload, and workflow issues • Consider your pedagogical needs, including resident learning, supervision and evaluation, and fragmentation of other rotations **Step 6 – Implement and monitor** • Inform all affected parties in a timely fashion before the schedule takes effect • Encourage local leaders to act as advocates for this culture change • Establish a system of active monitoring and feedback collection (including incident-driven review) prior to schedule implementation • Set a realistic timeline for evaluating and fine-tuning the system (avoid prematurely undertaking major changes or decisions) **Step 7 – Routine monitoring** • Continue monitoring for quality control after the initial changes • Develop institution-specific evaluation instruments for ongoing review of the new system ο Define purpose of review (quality control versus research) ο Determine and prioritize aspect(s) to be evaluated ο Identify stakeholders to be involved in the review ο Search for pre-existing evaluation instruments that may be adapted in your context ο Determine the most suitable methodology to conduct the evaluation (review the goal of the evaluation and balance resource requirements, scientific rigour, and level of relevance)

For those interested in assessing the impact of a new schedule with more rigour, the Kirkpatrick framework [58] is often used to evaluate the effect of a new training program or intervention. It divides all training outcomes into four levels of increasing importance: reaction, learning, behaviour, and results. In the case of a new duty hour system, examples of elements that are commonly assessed at the reaction level are residents’ evaluation of the new schedule. Residents’ performance on standardized tests can serve as a measure of learning. Outcomes at the behaviour level may be assessed using faculty and nurses’ evaluation of residents’ performance. Finally, patient safety measures such as morbidity and mortality statistics are examples of outcomes at the result level. As one progresses from one Kirkpatrick level of training outcome to another, the relevance increases, as do the number of confounders.

### Putting words into action: McGill’s experience

In order to illustrate how some of the concepts mentioned above have been operationalized in a large, university teaching hospital, we would like to describe our experience in designing and implementing a night float schedule for the Internal Medicine Residency Training Program at McGill University (Montreal, Quebec, Canada).

The McGill Internal Medicine Residency Training Program consists of a total of approximately 100 PGY-1 to PGY-3 residents distributed over three large teaching hospitals: the Jewish General Hospital (JGH), the Montreal General Hospital (MGH), and the Royal Victoria Hospital (RVH). The MGH and RVH together are part of the McGill University Health Centre (MUHC). Each hospital has an average of 10 to 15 residents at each PGY level. The MGH and RVH each have two 24-bed general medical Clinical Training Units (CTUs) and one 14-bed haematology/oncology CTU. The JGH has two medical CTUs, each consisting of 30 beds. These CTUs are geographically restricted, and patients are not under the care of the teams assigned to each CTU until a bed is available for the patient on one of the units.

In 2008, the Department of Medicine at the MUHC initiated and coordinated an external review of the CTUs. This review precipitated numerous changes to the CTUs at the MUHC, including a critical review of physician day and night staffing. The traditional model of physician staffing included a day team (a PGY-3, a PGY-2, 2 to 3 PGY-1s, and 2 to 3 medical students) and a night team (one of the on-call PGY-2s or PGY-3s and one of the on-call PGY-1s). The night teams covered all the medical CTUs at their hospital and admitted patients overnight. They also had primary responsibility for managing cardiac arrests. A separate team assessed and cared for medical patients in the emergency room at all three hospitals during both the day and night. Daytime teams would suffer from fragmentation as a result of residents being post-call, and patients covered by post-call residents would be redistributed to other members of the team, who would cover those patients for that day. Bedside teaching would suffer because of limited house-staff availability, depending on the team composition on a given day.

In light of this external review and feedback from residents and attending staff, and anticipating a regulatory framework change in the region regarding resident duty hour restrictions, the Department of Medicine at the MUHC transitioned to a model of care that integrated resident duty hour restrictions. Planning for this transition began in 2008, for a planned implementation on 1 July 2009. The planning committee included broad representation across the Department and training program, and focused on improving patient care and the teaching of both students and residents, as well as ensuring an appropriate workplace environment for attending staff. The committee recommended a move away from traditional call to a night float model.

In this model, the night float team consists of one PGY-3 and one PGY-1 who work from 8 p.m. to 8 a.m. This team has no daytime duties and are responsible for the same duties as the previous on-call team described above. In order to bridge the gap between when the daytime team leaves at 5 p.m. and when the night float team arrives at 8 p.m., we implemented an evening shift, which is covered by one senior and one junior resident until the night float team takes over. The night float team generally works five consecutive nights every seven days for one or two weeks in a row.

We opted for a phased implementation of this system, involving only PGY-3s from 2009 until 2010. During this period, we regularly sought feedback from residents, attending physicians, and nurses. As a result of this periodic review, we made various other adjustments to the day and night workflow in order to adapt to changing needs on the CTUs. Feedback from the PGY-3s was very positive, save for the feeling of isolation reported by seniors who felt disconnected from their PGY-1s, who were tired and wanted to go to bed overnight while the PGY-3s were interested in teaching. With successful implementation of this model at the PGY-3 level, we then proceeded to full implementation beginning in July 2010 by adding the PGY-1 residents across the MUHC, and also including the third teaching site (JGH). Feedback for this system has been very positive, with noted improvements in resident morale.

Working conditions for medical residents in Quebec, Canada, are governed provincially by a contract that applies to all residents working in the province. Given that the resident contract in Quebec – which came into effect on 1 July 2012 – incorporates resident duty hour restrictions, further adjustments to the critical care rotations are being piloted as this article goes to press. The models adopted for coverage in these units are variations on the shift and float models, with staggered shifts being implemented for units with the most acute and complex patients.

## Conclusion

With the advent of resident duty hour reform and a movement toward a 14- to 16-hour maximum consecutive work period, many institutions and programs are seeking to learn more about novel schedules. These scheduling systems generally incorporate the concepts of shifts and float teams. The choice will depend on context-specific clinical and pedagogical needs. It is likely that more than one type of scheduling model will co-exist within an institution, given the heterogeneity of patient and learner needs. Drawing on the experience of McGill University’s Internal Medicine Residency Training Program, we have outlined a practical approach to designing, implementing, and monitoring schedules that incorporates resident duty hour restrictions. Ultimately, the true challenges lie in facilitating acceptance of this culture change and in the systematic restructuring of clinical tasks to accommodate contemporary staffing models.

## Authors' contributions

Both authors contributed equally to the preparation of this paper.

## Competing interests

Ning Zi Sun was Chief Medical Resident during the implementation of night float at McGill University. Thomas Maniatis is the Program Director for the Internal Medicine Residency Training Program at McGill University. Both were involved with the design and implementation of night float as described in detail in this article.

## Declarations

Resources and secretariat support for this project was provided by the Royal College.

This article has been published as part of *BMC Medical Education* Volume 14 Supplement 1, 2014: Resident duty hours across borders: an international perspective. The full contents of the supplement are available online at http://www.biomedcentral.com/bmcmededuc/supplements/14/S1. This article was submitted peer reviewed in 2012. Final acceptance for publication as part of this supplement was in 2014.

Publication of this supplement was supported and funded by the Royal College of Physicians and Surgeons of Canada. The funding agency played no role in the design, in the collection, analysis and interpretation of data; in the writing of the manuscripts; and in the decision to submit the manuscript for publication. The articles have been through the journal's standard peer review process for supplements. The Supplement Editors declare that they have no competing interests.
